# Detection of SARS-CoV-2 Reinfections Using Nucleocapsid Antibody Boosting

**DOI:** 10.3201/eid3105.250021

**Published:** 2025-05

**Authors:** Eduard Grebe, Daniel Chacreton, Mars Stone, Bryan R. Spencer, James Haynes, Akintunde Akinseye, Marion C. Lanteri, Valerie Green, Hasan Sulaeman, Roberta Bruhn, Vivian I. Avelino-Silva, Paul Contestable, Brad J. Biggerstaff, Melissa M. Coughlin, Brian Custer, Jefferson M. Jones, David Wright, Michael P. Busch

**Affiliations:** Vitalant Research Institute, San Francisco, California, USA (E. Grebe, M. Stone, H. Sulaeman, R. Bruhn, V.I. Avelino-Silva, B. Custer, M.P. Busch); Westat, Rockville, Maryland, USA (D. Chacreton, A. Akinseye, D. Wright); University of California San Francisco, San Francisco (M. Stone, R. Bruhn, B. Custer, M.P. Busch); American Red Cross, Dedham, Massachusetts, USA (B.R. Spencer); American Red Cross, Rockville (J.M. Haynes); Creative Testing Solutions, Tempe, Arizona, USA (M.C. Lanteri, V. Green); San Francisco State University, San Francisco (H. Sulaeman); QuidelOrtho, Rochester, New York, USA (P. Contestable); Centers for Disease Control and Prevention, Fort Collins, Colorado, USA (B.J. Biggerstaff); Centers for Disease Control and Prevention, Atlanta, Georgia, USA (M.M. Coughlin, J.M. Jones)

**Keywords:** COVID-19, SARS-CoV-2, severe acute respiratory syndrome coronavirus 2, viruses, respiratory infections, zoonoses, serology, serosurveillance, blood donors

## Abstract

More than 85% of US adults had been infected with SARS-CoV-2 by the end of 2023. Continued serosurveillance of transmission and assessments of correlates of protection require robust detection of reinfections. We developed a serologic method for identifying reinfections in longitudinal blood donor data by assessing nucleocapsid (N) antibody boosting using a total immunoglobulin assay. Receiver operating characteristic curve analysis yielded an optimal ratio of >1.43 (sensitivity 87.1%, specificity 96.0%). When prioritizing specificity, a ratio of >2.33 was optimal (sensitivity 75.3%, specificity 99.3%). In donors with higher anti-N reactivity levels before reinfection, sensitivity was reduced. Sensitivity could be improved by expanding the dynamic range of the assay through dilutional testing, from 38.8% to 66.7% in the highest reactivity group (signal-to-cutoff ratio before reinfection >150). This study demonstrated that longitudinal testing for N antibodies can be used to identify reinfections and estimate total infection incidence in a blood donor cohort.

In earlier phases of the COVID-19 pandemic, cross-sectional serosurveillance was informative for establishing cumulative incidence rates and the prevalence of previous infection in a population (*1*). In countries with spike (S)-based vaccines, previous SARS-CoV-2 infection could be detected using anti-nucleocapsid (N) serologic assays, which in combination with anti-S assays could discriminate vaccine-induced antibody reactivity from infection-induced antibody reactivity (*2*). However, because the epidemic evolved with increasing seroprevalence and most of the global population have experienced >1 SARS-CoV-2 infections, using serologic methods to estimate infection incidence now requires robust detection of reinfections. Furthermore, after the public health emergency declaration expired, case reporting and collection for public health surveillance decreased, limiting the ability to monitor transmission and disease burden, particularly rates of asymptomatic infection and subclinical reinfection.

The National Blood Donor Cohort (NBDC) is a longitudinal study of blood donors sponsored by the US Centers for Disease Control and Prevention and conducted in partnership with the 2 largest US blood collectors, Vitalant and the American Red Cross; their central testing laboratory, Creative Testing Solutions; and Westat (*3*). An earlier iteration of this program, the National Blood Donor Serosurvey, executed serial monthly cross-sectional serosurveys during July 2020–December 2021 (*1*,*4*–*7*) to provide population-weighted seroprevalence estimates. By the end of 2021, the proportion of donation specimens with vaccine-induced or infection-induced anti-S seroprevalence approached 95%, and infection-induced anti-N seroprevalence approached 30% (*6*). Because reinfections were known to become more common beginning in 2022 (*8*–*10*), we modified the blood donor study to a longitudinal design to enable detection of reinfections. Longitudinal testing is required to identify reinfections through boosting of infection-induced antibodies. Here, we describe the methods developed to detect reinfections in blood donors by detecting boosting of N antibodies, and measure the performance of those methods.

## Materials and Methods

### Study Population

Blood donors with a history of regular blood donation and with known prior SARS-CoV-2 infection and COVID-19 vaccination status (determined during the June 2020–June 2021 screening period) were selected for continued monitoring in the NBDC. Eligible donors were those who sought to donate blood at least twice during the screening period and met all blood donor eligibility criteria. The NBDC included 142,599 donors who were categorized into 4 groups by previous SARS-CoV-2 infection and vaccination status as of mid-2021. We established groups by testing donation specimens with the VITROS Anti-SARS-CoV-2 S total immunoglobulin (Ig) assay (QuidelOrtho, https://www.quidelortho.com) and Elecsys Anti-SARS-CoV-2 N total Ig assay (Roche, https://www.roche.com), as well as self-reported COVID-19 vaccination status (*1*,*3*,*6*,*11*). During follow-up, July 2021–December 2022, we identified donation specimens from donors in the cohort in real time and stored those specimens frozen. In 2022, we typically tested 1 donation specimen per donor per quarter (if the donor presented in that quarter), using VITROS Anti-SARS-CoV-2 IgG Quantitative test (Ortho anti-S IgG; QuidelOrtho) and VITROS Anti-SARS-CoV-2 Total N Antibody assay (Ortho anti-N total Ig; QuidelOrtho) at Creative Testing Solutions and Vitalant Research Institute. For certain substudies, more frequent longitudinal samples were tested.

Self-reported vaccination status was captured at each donation as part of routine donation procedures (M. Stone, unpub. data). We invited all cohort donors to respond to quarterly electronic surveys to report vaccination and infection history, including date and manufacturer of vaccine doses, which were not collected routinely at donation. Survey data enabled identification of swab-confirmed or physician-diagnosed first infections and reinfections, and associated symptoms and clinical outcomes. The overall survey response rate was 46.5%. We restricted this study to survey respondents with informative survey responses, i.e., responses that followed tested donation specimens.

### Definition of Cases and Controls for Identifying Anti-N Boosting Criteria

We defined confirmed reinfections (cases) as survey-reported swab-confirmed reinfections. Methods for confirmation were a viral test, such as a rapid antigen test or laboratory-based PCR test, or a physician diagnosis (presumed to be on the basis of diagnostic testing). The first infections before the confirmed reinfections could be serologically identified by anti-N seroconversion or be reported as swab-confirmed infections. To classify a swab-confirmed infection as a reinfection, the reinfection had to occur >90 days after either seroconversion or a previous swab-confirmed infection. We identified a total of 2,681 cases of swab-confirmed reinfection.

We identified donors from early in the pandemic (the second half of 2020), when reinfections were rare (*12*). We selected donors for whom we had >2 longitudinal anti-N results, the first of which had been >56 days after seroconversion. Among those donors, we defined controls as donors who responded to the electronic survey and did not report any swab-confirmed or suspected infections during relevant interdonation intervals (IDIs). We identified a total of 5,150 controls.

### Laboratory Testing and Anti-N Reactivity Trajectories

We tested donation specimens from cases and controls with the Ortho anti-N total Ig assay in accordance with the manufacturer’s instructions. That semiquantitative assay reports signal-to-cutoff (S/CO) ratios, which we used to calculate changes in anti-N reactivity. The assay has high sensitivity to detect first infections in vaccinated (98.2% sensitivity) and unvaccinated (95.6% sensitivity) persons (*13*).

After initial results indicated insufficient dynamic range to detect boosting in persons who had high anti-N reactivity (S/CO >100), we developed a dilutional testing algorithm to extend the dynamic range of the assay. The algorithm implemented a 2-step dilution procedure: if the undiluted specimen (neat testing) yielded an S/CO >100, we retested the specimen in a 1:20 dilution. If the S/CO yielded by the 1:20 dilution (before multiplication) was still >100, we further tested the sample in a 1:400 dilution. We programmed and performed those dilutions on the VITROS instrument as reflex testing. The final estimated S/CO (reactivity) of the sample was then the S/CO obtained from the final dilution (neat, 1:20, or 1:400) multiplied by the dilution factor (1, 20, or 400).

We plotted individual donors’ anti-N trajectories, and derived average trajectories across all donors included in the analysis after first infection, before reinfection, and after reinfection. We stratified average trajectories by vaccination status.

### Identifying Anti-N Boosting Criteria

We evaluated 2 methods to detect anti-N boosting for sensitivity and specificity. First, we estimated the slope in reactivity between 2 donation specimens (difference in log S/CO obtained on subsequent samples divided by time elapsed between samples). If the slope was positive (indicating an increase in reactivity) and exceeded a set threshold, we classified the IDI as a reinfection. We used the identified cases and controls for identifying optimal slope and ratio thresholds and to assess the performance of thresholds. We included only first reinfection (i.e., second infection) cases in the analysis.

Second, we derived a ratio of anti-N reactivity at the end of the IDI to reactivity at the start of the IDI, and if the ratio exceeded a threshold, we classified it as a reinfection. The first approach has the theoretical advantage over the second of accounting for lengths of IDIs, which are highly variable, but has the disadvantage of being more complicated to calculate.

Time to peak anti-N reactivity after first infections is variable. Misclassification can result from computing a ratio using sequential values observed during the initial ramp-up phase of anti-N reactivity after a first infection. That misclassification could result in an apparent reinfection-associated boost when in fact it represents continuing antibody reactivity increase (maturation) associated with 1 infection. For those reasons, we refined the method to only consider IDIs eligible for reinfection detection when the first specimen was collected >56 days after initial seroconversion (i.e., after first observed anti-N reactive donation). We chose the cutoff of 56 days on the basis of reported peak anti-N at 30–90 days after symptom onset, although that peak can be influenced by disease severity (*14*). Our reason was that a 56-day minimum was likely to reduce misclassification because of maturing antibody responses after first infection, while retaining most whole-blood donors for whom a minimum interdonation interval of 56 days applied. Furthermore, because very low S/COs can be unstable, and because very small absolute increases might exceed identified ratio thresholds, we set S/COs <1 to 1 for the purpose of calculating the ratio (Appendix). We imposed no maximum IDI length.

### Statistical Analysis

#### Identification of Optimal Boosting Thresholds

We used ROC curve analysis to identify optimal anti-N boosting thresholds for detection of first reinfections, using the 2 approaches to quantify boosting we described. We defined optimality in 2 ways: first, on the basis of an equal weighting of sensitivity and specificity, as the threshold that maximized Youden’s J statistic. Second, given that poor specificity would severely affect population-level estimates in a context in which reinfections are relatively rare, we defined optimality based on a weighted Youden’s J, which prioritized specificity (Appendix). We chose the weight on the basis of the conservative assumption that 1 in 40 infections are reinfections. We therefore identified 2 sets of optimal thresholds, based on Youden’s J and weighted Youden’s J, for both the slope and ratio methods of classification. The weight could be adjusted or abandoned for later studies conducted when reinfections represented a larger proportion of all infections. Beyond sensitivity and specificity, we further evaluated only the ratio method in this analysis, given similar performance and reduced complexity. To further assess performance of anti-N boosting thresholds, we computed positive and negative predictive values (PPVs and NPVs) under different scenarios defined by hypothetical rates of reinfection.

#### Effect of Prereinfection Anti-N Reactivity Level on Sensitivity and Specificity

To assess the effect of assay saturation (limited dynamic range suppressing higher S/COs, which potentially limited the ability to observe reinfection-associated boosting), we stratified cases and controls by the anti-N S/CO at the start of the IDI and computed sensitivity and specificity using the thresholds derived from Youden’s J and weighted Youden’s J in each stratum. The strata were S/CO <50, >50 to <100, >100 to <150, and >150. We further computed the median ratio observed in cases and controls in each stratum.

#### Sensitivity of Dilutional Anti-N Testing for Detection of First Reinfections

Because we performed dilutional testing only on a subset of cases, and not controls, we could assess performance only in terms of sensitivity, which was the parameter affected by the reactivity level at the start of the interval used to compute ratios. We computed the sensitivity (using both thresholds) in each stratum for the neat (undiluted) testing–only algorithm and the dilutional-testing algorithm. We further report the median observed ratios in cases for each testing algorithm. We conducted all analyses using SAS version 9.4 (SAS Institute Inc., https://www.sas.com) and Python version 3.10.11 (Python Software Foundation, https://www.python.org). 

## Results

### Anti-N Boosting Associated with Reinfection

**Figure 1:**
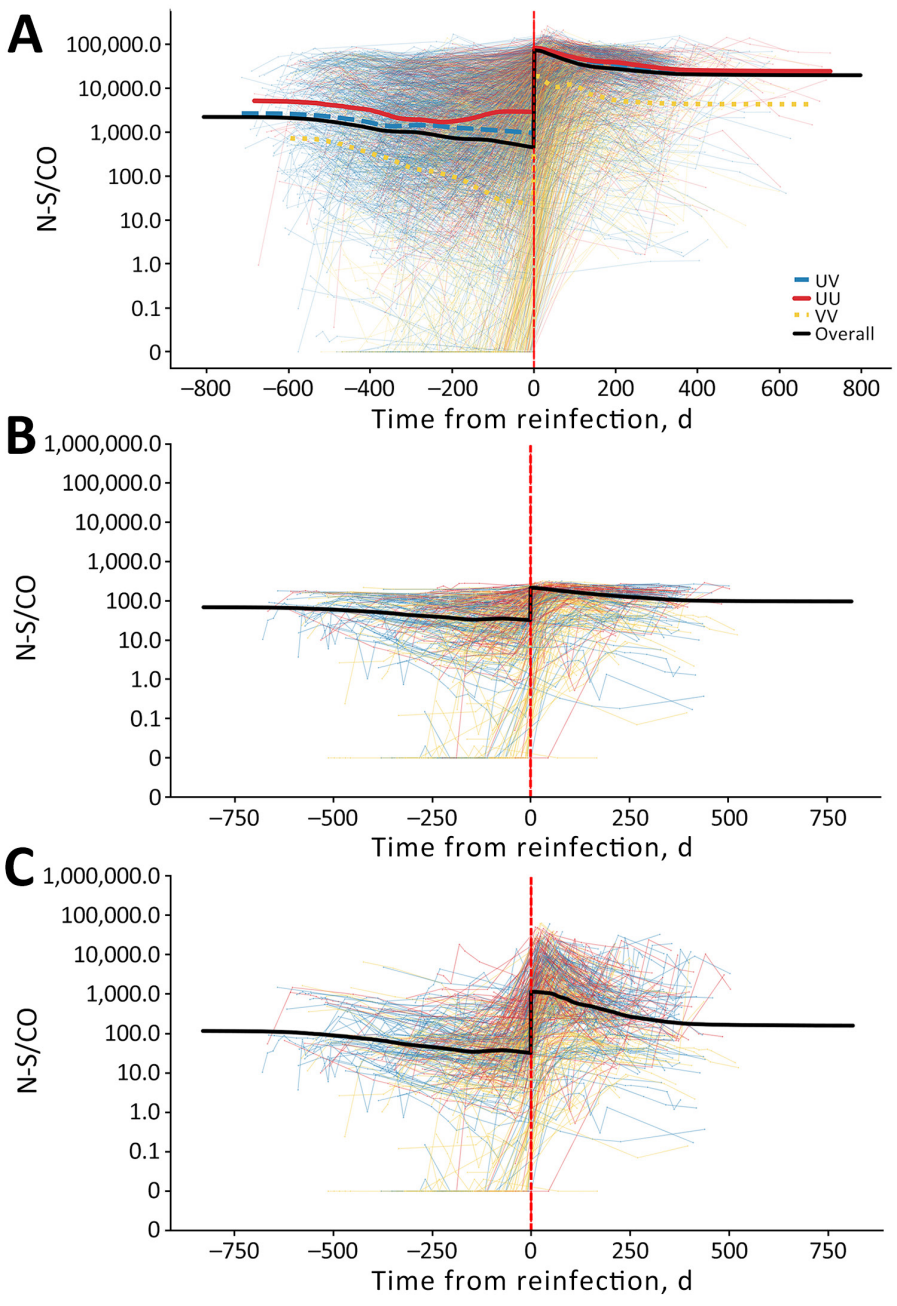
Individual anti-N S/CO trajectories before and after swab-confirmed reinfection in vaccinated and unvaccinated participants in study of detection of SARS-CoV-2 reinfections using nucleocapsid antibody boosting. A) S/CO trajectories with neat-only anti-N testing of all donors with reinfections in the study. B) Trajectories of test results from 434 donors with reinfections subjected to expanded dynamic range dilutional anti-N testing; neat results only. C) Trajectories of test results from the same 434 donors with reinfections subjected to dilutional anti-N testing; dilutional (expanded dynamic range) testing results only. Images show average anti-N trajectories of donors who experienced reinfections, with and without expanded dynamic range testing, stratified by vaccination status. Time represents days before or after swab-confirmed reinfection (vertical red dashed line). N, nucleocapsid; S/CO, signal-to-cutoff ratio; UU, unvaccinated at the time of first infection and reinfection; UV, unvaccinated at first infection and vaccinated at reinfection; VV, vaccinated at first infection and reinfection.

In donors reporting swab-confirmed reinfections, individual neat anti-N reactivity trajectories tended to wane slowly or remain stable after first infections with clear boosting of antibody reactivity after reinfection (Figure 1). As previously reported (*15*), vaccination history affected the level of reactivity observed; unvaccinated donors exhibited higher postinfection anti-N reactivity than vaccinated donors. Donors who were vaccinated before their first infection showed overall lower reactivity levels than unvaccinated donors, and donors who were vaccinated between the 2 infection events showed reactivity that fell between donors who were vaccinated before the first infection and those who were not vaccinated (Figure 1, panel A). However, the relative magnitude of the anamnestic boost induced by reinfection was similar across vaccination groups.

### Identification of Optimal Boosting Thresholds

The optimal anti-N boosting threshold for the slope approach, using the unweighted Youden’s J method, was >0.003 log_10_(S/CO) per day, and using the weighted Youden’s J method was >0.006 log_10_(S/CO) per day. Using unweighted Youden’s J method, sensitivity was 87.2% and specificity was 97.0%. Using weighted Youden’s J method sensitivity was 78.8% and specificity was 99.4% (Table 1). For the simpler ratio approach, the optimal thresholds were >1.43 (95% CI 1.27–1.61) for the unweighted Youden’s J method and >2.33 (95% CI 2.12–2.56) for specificity-prioritized weighted Youden’s J method. For unweighted Youden’s J method, associated sensitivity was 87.1% and specificity was 96.0%; for specificity-prioritized weighted Youden’s J method, sensitivity was 75.3% and specificity was 99.3% (Table 1).

**Table 1:** Reinfection classification data determined from receiver operating characteristic curve analysis in study of SARS-CoV-2 reinfections detected by nucleocapsid antibody boosting*

Classification method	Statistic†	Optimal threshold (95% CI)	Sensitivity, % (95% CI),‡ n = 2,681	Specificity, % (95% CI),‡ n = 5,150
Pre–/post–anti-N slope	Unweighted Youden's J	0.003 (0.003–0.004)	87.24 (86.09–87.84)	96.97 (96.04–97.61)
Pre–/post–anti-N slope	Weighted Youden's J	0.006 (0.006–0.007)	78.78 (76.73–80.16)	99.38 (99.18–99.38)
Post–/pre–anti-N ratio	Unweighted Youden's J	1.43 (1.27–1.61)	87.09 (84.48–88.77)	95.96 (93.24–97.51)
Post–/pre–anti-N ratio	Weighted Youden's J	2.33 (2.12–2.56)	75.31 (72.7–77.88)	99.34 (99.13–99.38)

*N, nucleocapsid. †Weighted method prioritized specificity by applying a weight to specificity (see Methods). ‡Sensitivity and specificity associated with lower and upper CI limits on optimal threshold.

### Seroconversion in Anti-N Negative Prereinfection Samples

Of 2,681 swab-confirmed reinfection cases, 328 (12.2%) did not demonstrate anti-N reactivity above the threshold for positivity (S/CO >1) at the immediate prereinfection sample. Of those that did not demonstrate anti-N reactivity, 296 were available for further evaluation; 246 (83.1%) had never seroconverted after the first reported infection and 50 (16.9%) seroreverted before reinfection. All 328 donors with negative prereinfection results seroconverted after reinfection (Figure 1).

### Effect of Post–First Infection Anti-N Reactivity on Performance of Anti-N Boosting Thresholds

Using the lower threshold of the ratio method derived from the unweighted Youden’s J method, we noted that as prereinfection S/CO increased, sensitivity declined from 93.8% in the S/CO <50 group to 88.0% in the S/CO >100–150 group and was lowest at 38.8% in the S/CO >150 group. Specificity was similar across prereinfection reactivity strata (91.7% to 97.0%) (Table 2). Using the higher threshold derived from the weighted Youden’s J method, we noted that sensitivity declined from 91.6% in the S/CO <50 group to 0.8% in the S/CO >150 group. Specificity was similar across prereinfection reactivity strata (98.9% to 100.0%). Median observed postreinfection to prereinfection S/CO ratios in cases also declined in higher prereinfection reactivity strata, from 15.2 in the lowest reactivity stratum to 1.3 in the highest reactivity stratum (Table 2).

**Table 2:** Reinfection classification data determined from ratio approach in study of SARS-CoV-2 reinfections detected by nucleocapsid antibody boosting*

	Cases			Controls			Unweighted Youden's J (1.43)			Weighted Youden's J (2.33)	
S/CO	No.	Median ratio		No.	Median ratio		Sensitivity, %	Specificity, %		Sensitivity, %	Specificity, %
Overall	2,681	6.22		5,150	0.55		87.1	96.0		75.3	99.3
0–50	1,661	15.2		2,237	0.49		93.8	97.0		91.6	98.9
50–100	485	3.41		1,042	0.49		89.5	97.3		81.9	99.2
100–150	275	2.15		757	0.56		88.0	91.7		35.6	99.9
>150	260	1.28		1,114	0.76		38.8	95.5		0.8	100.0

*S/CO value is prereinfection. Unweighted and weighted boosting thresholds are ratios of postreinfection to prereinfection S/CO. S/CO, signal-to-cutoff ratio.

### Dilutional Anti-N Testing to Improve Detection of Reinfections

**Figure 2:**
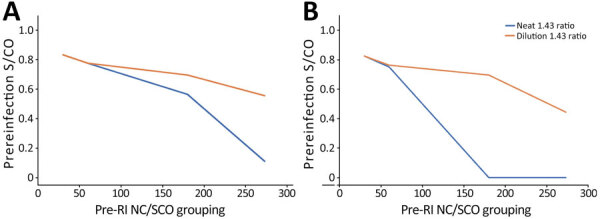
Effect of prereinfection anti-N S/CO on performance of boosting thresholds in study of detection of SARS-CoV-2 reinfections using nucleocapsid antibody boosting. A) Sensitivity by prereinfection anti-N S/CO using neat and dilutional testing for the unweighted threshold (>1.43); B) sensitivity by prereinfection anti-N S/CO using neat and dilutional testing for the weighted threshold (>2.33). S/CO, signal-to-cutoff ratio.

We tested a subset of cases using the anti-N dilutional algorithm (n = 434). When we used the neat testing results only, sensitivities were 85.1% in the group with prereinfection S/CO <50 and 38.5% in the S/CO >150 group when using the unweighted ratio threshold and 84.4% in the S/CO <50 and 0.0% in the S/CO >150 group when using the weighted ratio threshold (Table 3). Dilutional testing improved sensitivity in the group with prereinfection S/CO >150 to 66.7% for the unweighted ratio thresholds, and to 61.5% for weighted ratio thresholds. For groups with prereinfection S/CO 100–150, sensitivity remained >80% for weighted and unweighted ratio thresholds when we performed dilutional testing (Table 3; Figure 2).

**Table 3:** Reinfection detection data by prereinfection anti-N reactivity level in study of SARS-CoV-2 reinfections detected by nucleocapsid antibody boosting*

S/CO†	No. cases		Median ratio			Sensitivity of unweighted Youden's J (1.43)			Sensitivity of weighted Youden's J (2.33)	
			Neat	Dilution		Neat, %	Dilution, %		Neat, %	Dilution, %
Overall	434		4.50	58.54		79.3	81.6		65.0	80.6
0–50	262		10.94	94.32		85.1	85.1		84.4	84.4
50–100	78		2.82	64.97		78.2	78.5		69.2	78.2
100–150	55		1.90	29.49		81.8	80.0		12.7	80.0
>150	39		1.35	9.20		38.5	66.7		0.0	61.5

*Reactivity was measured with and without expanded dynamic range anti-N testing reinfection cases. S/CO, signal-to-cutoff ratio. Unweighted and weighted boosting thresholds are ratios of postreinfection to prereinfection S/CO. †S/CO value is prereinfection.

### Performance of Anti-N Boosting Thresholds at Different Population Reinfection Rates

**Figure 3:**
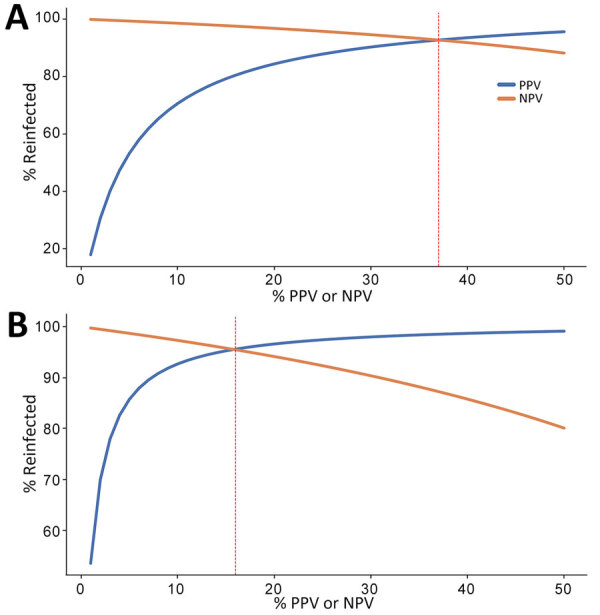
PPV and NPV in a study of detection of SARS-CoV-2 reinfections using nucleocapsid antibody boosting. A) Unweighted threshold ratio >1.43. B) Weighted threshold ratio >2.33. Predictive values were calculated as a function of percentage of a population of blood donors experiencing SARS-CoV-2 reinfection, at different rates of reinfection. Vertical red lines indicate the proportions reinfected that represent the optimal scenarios for the given threshold ratio, i.e., where PPV and NPV are simultaneously maximized. NPV, negative predictive value; PPV, positive predictive value.

When the percentage of population experiencing reinfection was low, NPV was high and declined slowly as the rate of reinfection increased, and PPV was low but increased rapidly as the reinfection rate increased. PPV >80% was achieved when the lower ratio threshold was >15% of the population reinfected (Figure 3, Panel A), and when the higher ratio was >5% of the population reinfected (Figure 3, panel B). The optimal scenario for our thresholds (maximizing PPV and NPV) were 37% reinfected for the lower ratio threshold and 16% reinfected for the for the higher ratio threshold.

## Discussion

We evaluated a method for serologic identification of reinfections using anamnestic boosting of anti-N reactivity in longitudinal blood donor samples. Anti-N boosting thresholds optimized to maximize specificity achieved reasonable sensitivity (>75%) and excellent specificity (>99%) for detection of first reinfections. We also derived a lower threshold that achieves sensitivity >87%, but sacrifices some specificity. However, the sensitivity to detect first reinfections was quite low in donors with high anti-N reactivity after first infections; reactivity plateaued near the top of the assay’s limited dynamic range and masked anamnestic boosting associated with reinfections. Thus, we developed a dilutional testing algorithm that dramatically expanded the dynamic range, greatly improving sensitivity to detect reinfections in persons with high anti-N reactivity before reinfection. We based the trigger for dilutions (S/CO >100) on guidance from the manufacturer, who conducted studies to identify the linear dilutional performance range (P. Contestable, pers. comm., confirmed by email 2025 Apr 7). As multiple reinfections become increasingly common, expanded dynamic range testing will become increasingly important. 

We could not assess the specificity of the dilutional algorithm on the basis of the reflex testing criteria used in this study because we did not perform dilutional testing of controls; the risk for reduced specificity (as demonstrated in neat specimen reactivity ratios) suggests using the higher threshold (ratio >2.33) in expanded dynamic range testing. Although sensitivity in the neat testing dropped to 0% using the higher threshold for donors with anti-N S/CO >150 before reinfection, sensitivity was maintained at >60% for this group with dilutional testing.

We considered multiple methods for identifying reinfections based on anti-N boosting. The first and most complex relied on estimating an individual postinfection anti-N reactivity waning rate on the basis of >2 observations after first infection. That waning rate would then be used to estimate an expected value of anti-N reactivity at the time of a later donation specimen, and the expected value compared to the observed reactivity. That approach would have enabled us to incorporate uncertainty in expected reactivity arising from assay variability, inconsistent waning patterns, or other factors. We could then compare the observed anti-N reactivity to the expected reactivity; if the former exceeded the latter by a set threshold (e.g., 2 SD), we would classify the IDI as one in which a reinfection occurred. We abandoned that approach because of its complexity, highly variable time to peak and peak level of reactivity time after first infections, relative stability in anti-N reactivity in the Ortho assay, and difficulty in robustly estimating individual waning rates.

Although we did not pursue the originally envisaged method based on estimating average and person-specific anti-N waning rates, that approach could be further explored, especially if an IgG assay is used; IgG assays tend to show more rapid waning than total Ig assays (*2*,*16*). The method and thresholds identified as optimal in this study apply specifically to the Ortho Total Ig anti-N assay. The rapid waning of IgG assays may have advantages for detection of reinfection-associated antibody boosting, although the length of time intervals between specimens would also be important for interpretation.

We did not expect the finding that 12.2% of prereinfection samples had anti-N S/CO below the threshold for positivity. Possible causes are misreporting of infections or infection dates, seroreversion, or a failure to develop anti-N antibodies after the first infection. We previously reported that 1.9% of samples from unvaccinated donors and 4.4% collected from vaccinated donors after first swab-confirmed infections tested anti-N nonreactive on the Ortho assay (*14*). Petersen et al. (*17*) also reported similar rates of failure to develop antibodies after SARS-CoV-2 infection. All donors seroconverted after the reported reinfection, although serology alone would classify those infections as first infections.

A limitation of our study is that we relied on self-reported infection and vaccination history, supported by our serologic testing. Furthermore, we could not establish with certainty that our (nonreinfected) controls for the ROC curve analysis had not experienced reinfections. However, the contamination of our results by controls who did experience reinfections during the intervals used in the analysis is likely minimal because reinfections were very rare at the time that the donation specimens were collected (*12*). Cases were all swab-confirmed, and because testing is usually triggered by symptomatic disease, cases largely represent symptomatic reinfection cases. Therefore, our thresholds might not be accurate for detection of asymptomatic reinfections; accuracy is further complicated by the possibility of exposures that do not result in substantial viral replication and consequently do not trigger an anamnestic boosting of antibodies (*18*). A further limitation is that no expanded dynamic range testing was available on controls because of limited testing capacity; therefore, we could not identify optimal thresholds for a dilutional testing regime. Second, third, and subsequent reinfections are not included in this study but are the subject of future work. Finally, blood donors are not demographically representative of the general population, and the healthy donor effect means that chronic health conditions are less prevalent in blood donors than in the general population (*19*,*20*).

Despite those limitations, blood donor cohorts have tremendous value for public health research, including enabling serosurveillance of infectious diseases in a healthy population, related focused studies such as correlates of protection and population immunity studies, rapid response to emerging infectious threats, and the ability to address a wide range of general health-related questions in a low-cost manner. The platform established by the NBDC has been leveraged by the Centers for Disease Control and Prevention and its partners to develop a broader respiratory virus surveillance program.

In conclusion, we developed and measured the performance of a method for detecting boosting of N antibodies to identify SARS-CoV-2 reinfections. The method enables detection of total infection incidence by combining detections of first-time infections through anti-N seroconversion with detection of reinfections. Given that most persons have previously been infected with SARS-CoV-2 and public health case reporting has decreased, methods to detect reinfections are needed to estimate the burden of COVID-19 moving forward. Seroepidemiology can provide specific estimates of infections, complementing trends in wastewater surveillance, and COVID-19 test positivity. In addition, antibody testing enables assessment of correlates of protection and vaccine effectiveness against mild or asymptomatic infections.

Appendix Mathematical formulas used in study of detection of SARS-CoV-2 reinfections using nucleocapsid antibody boosting.
