# Supplementary material for: Detection of SARS-CoV-2 Reinfections Using Nucleocapsid Antibody Boosting
Source: Emerg Infect Dis. 2025 May;31(5):958–66. doi: 10.3201/eid3105.250021 (PMC12044254; doi:10.3201/eid3105.250021)
Supplement: Appendix — Mathematical formulas used in study of detection of SARS-CoV-2 reinfections using nucleocapsid antibody boosting. [file 25-0021-Techapp-s1.pdf]

Article DOI: https://doi.org/10.3201/eid3105.250021

EID cannot ensure accessibility for supplementary materials supplied by authors. Readers who have difficulty accessing supplementary content should contact the authors for assistance.

Detection of SARS-CoV-2 Reinfections Using Nucleocapsid Antibody Boosting

Appendix

Mathematical Formulas

The ratio of anti-nucleocapsid reactivity (signal-to-cutoff ratio, S/CO) was calculated as follows:

$$ratio = \frac{S/CO_{t_2}}{\max\,(1, S/CO_{t_1})}$$

with S/CO_{t_1} the S/CO at the start and S/CO_{t_2} the S/CO at the end of the interdonation interval.

The unweighted and weighted Youden's J were calculated using the formulas below:

$$J = Sensitivity + Specificity - 1$$

$$J_W = 2(W \cdot Sensitivity + (1 - W) \cdot Specificity) - 1$$

with J_W the weighted Youden's J, and the weight $W \leq 1$.
